# T Helper Cell Subsets in Clinical Manifestations of Psoriasis

**DOI:** 10.1155/2016/7692024

**Published:** 2016-08-10

**Authors:** Marco Diani, Gianfranco Altomare, Eva Reali

**Affiliations:** ^1^I.R.C.C.S Istituto Ortopedico Galeazzi, Department of Dermatology and Venereology, University of Milan, 20161 Milan, Italy; ^2^I.R.C.C.S Istituto Ortopedico Galeazzi, Laboratory of Translational Immunology, 20161 Milan, Italy

## Abstract

Psoriasis is a chronic inflammatory skin disease, which is associated with systemic inflammation and comorbidities, such as psoriatic arthritis and cardiovascular diseases. The autoimmune nature of psoriasis has been established only recently, conferring a central role to epidermal CD8 T cells recognizing self-epitopes in the initial phase of the disease. Different subsets of helper cells have also been reported as key players in the psoriasis pathogenesis. Here, we reviewed the knowledge on the role of each subset in the psoriatic cascade and in the different clinical manifestations of the disease. We will discuss the role of Th1 and Th17 cells in the initiation and in the amplification phase of cutaneous inflammation. Moreover, we will discuss the recently proposed role of tissue resident Th22 cells in disease memory in sites of recurrent psoriasis and the possible involvement of Th9 cells. Finally, we will discuss the hypothesis of a link between T helper cell subsets recirculating from the skin and the systemic manifestations of psoriasis.

## 1. Introduction

Psoriasis is a chronically relapsing hyperproliferative skin inflammation affecting about 2% of the population worldwide and is among the most frequent T cell-mediated disorders. Plaque psoriasis, also known as* psoriasis vulgaris*, is the commonest form of the disease, accounting for almost 90% of psoriatic patients [1, 2].

Histologically, psoriasis is characterized by three principal features: epidermal hyperplasia, leukocyte infiltrates, and an increased number of tortuous and leaky vessels in the dermis resembling the regenerative maturation observed during wound repair. In recent studies, we and others have reported the enhanced presence of lymphoid aggregates in the dermis of psoriatic plaques as compared to normal skin [3, 4].

About 30% of patients with psoriasis also develop psoriatic arthritis, characterized by synovitis, enthesitis, and dactylitis. Besides skin and joint inflammation there is evidence that psoriasis is associated with important systemic manifestations and with comorbidities such as cardiovascular disorders, metabolic syndrome, and Crohn's disease [5–7].

Many advances have been made in the last decades in the understanding of psoriasis pathogenesis leading to the identification of the T cell subsets that play a role in establishing inflammation in psoriatic skin lesions.

Different subsets of T cells may have a different hierarchical role in the pathogenesis of the disease underlining the importance of distinguishing T cell subpopulations involved in the initiation phase of the disease from those acting as downstream effectors amplifying inflammation in psoriatic plaques possibly accounting for the extent of the clinical manifestations [2, 8].

The autoimmune nature of psoriasis has been established only recently by the discovery of the role of cathelicidin (LL37), keratinocyte-derived antimicrobial peptide, as an autoantigen for both CD8 and CD4 autoreactive T cells in psoriasis patients.

In addition, a second antigen, ADAMTS-like protein 5 (ADAMTSL5), which is produced by melanocytes, has also been identified as an autoantigen recognized by IL-17-producing CD8 T cells, restricted by HLA-C^*∗*^06:02 [9, 10].

These two reports together with early studies on self-reactive CD8 T cells strongly support that CD8 T cells represent the autoimmune core of the disease driving the initial phase of psoriasis and repository of specific disease memory [11–15].

As regards T helper cells, in the last several years, different subsets have been described as critical factors in psoriasis pathogenesis. Specifically, Th1 cells that were originally considered as the main player in psoriasis pathogenesis have been proposed to be actually more relevant in early stages of the disease, upstream of the IL-17 driven proinflammatory loop. At the same time, genetic association studies together with human and mouse experimental data and the results of clinical trials have shifted the attention to IL-23/Th17 axis controlling mainly the proinflammatory loop in psoriatic plaques which involves keratinocytes, dendritic cells, and T cells [16, 17]. It is important to mention that *γδ* T cells also play a major role in the production of IL-17 and in the maintenance of inflammation in psoriatic plaques [2, 18–22].

More recently, the role of tissue resident Th22 cells in psoriasis has been enlightened and a role for Th9 cells has been proposed.

We have recently reported that CCR4^+^ CD4^+^ T cells are increased in the circulation of patients with psoriasis proportionally with the increased severity of the cutaneous manifestations of the disease expressed as PASI (Psoriasis Area and Severity Index) score. This links CD4^+^ T helper cells recirculating between skin and blood with the clinical manifestations of psoriasis.

## 2. Th17 and Th1: Key Player in Psoriasis Pathogenesis

Psoriasis was originally considered a Th1-mediated skin disease, whereas, in recent years, the focus has been shifted to Th17 cells and to other IL-17-producing cell types, notably *γδ* T cells and CD8 T cells [23–27].

Th17 cytokines, in particular interleukin-17A (IL-17A), have been shown to have a pivotal role in sustaining inflammation in psoriatic plaques. The number of IL-17-producing CD4^+^ T cells has been observed to be much higher in psoriatic skin lesions than in healthy skin [28–30]. Th17 cells are crucial for the secretion of IL-17A, IL-17F, IL-22, and IL-9, which promote the inflammatory response of keratinocytes as indicated by studies in cultured human keratinocytes and in an IL-17-treated reconstituted human epidermis model [31]. Antimicrobial peptides, cytokines, and chemokines such as CCL20 and CXCL1, CXCL3, and CXCL8 are part of the IL-17-induced signature in keratinocytes and play a crucial role in amplifying the immune response in psoriatic plaques [31–35]. Therefore, activated Th17 cells can enhance the inflammatory response of keratinocytes by creating a positive feedback loop around the IL-23/Th17 axis [16].

The key role of this axis in psoriasis pathogenesis is strongly supported by the association between IL-23R, IL-12B, and IL-23A gene variants and psoriasis susceptibility [36]. Definitive evidence finally emerged from the results of clinical trials with anti-IL-17 antibody showing very rapid and highly effective outcome in patients with cutaneous psoriasis. In these trials, treatments with secukinumab and ixekizumab, anti-IL-17 monoclonal antibodies, and brodalumab, an IL-17RA receptor antagonist, indicate IL-17 inhibitors as superior in psoriasis efficacy compared to TNF*α* inhibitors and IL-12/IL-23 inhibitors with very few serious adverse events [37–43]. The rapid and highly effective results of anti-IL-17-based therapy strongly support the concept of a role for IL-17 as a pivotal amplifying effector mechanism in psoriasis determining the extent of the cutaneous manifestations of the disease [44].

As a consequence of the strong evidence of the central role of IL-17-producing cells, the relevance of Th1 cells and IFN*γ* has become less clear. In lesional skin of psoriasis patients Th1 cells and IFN*γ* levels are clearly increased [45, 46]. Results from a study by Kryczek et al. have suggested that IFN*γ* exerts one of its main effects in psoriasis pathogenesis upstream of IL-17 in the psoriatic cascade. According to this view IFN*γ* produced by Th1 and other cells would program myeloid dendritic cells to produce CCL20 ligand of CCR6 and to secrete IL-23. This in turn would favor the recruitment and expansion of IL-17-producing cells. IFN*γ* mRNA is indeed markedly upregulated in psoriatic plaques and noticeably genes which in keratinocytes are induced by IFN*γ*, such as* CXCL9, CXCL10*, and* CXCL11*, are also upregulated in psoriatic lesions. This evidence could point towards a role for IFN*γ* in mediating further recruitment of CXCR3^+^ Th1 and CD8 T cells in psoriatic plaques [35, 47, 48]. This is in line with our recent data indicating recruitment of CCR5^+^ IFN*γ*-producing CD4^+^ T cells to the psoriatic plaques at advanced stages of the disease [49].

On the other hand, in mouse models of autoimmune diseases such as Collagen-Induced Arthritis (CIA) and induced experimental autoimmune encephalomyelitis (EAE), it has been shown that IFN*γ* suppresses the production of IL-17 and, in the case of CIA, it exerts a protective effect against the disease development [50–52].

Moreover, in a small pilot study, humanized anti-IFN*γ* antibody has been used in the treatment of psoriasis showing only a minor therapeutic effect [53, 54].

These controversial findings underline a complex interplay between Th1 and Th17 cells in the pathogenesis of psoriasis and despite the marked enhancement of both cytokines in psoriatic skin lesions the understanding of their relative role in disease pathogenesis needs to be further clarified.

## 3. Th22

Th22 cells are important for skin homeostasis and in inflammation. Similarly to Th17, they express IL-23R but mainly produce IL-22 in the absence of IL-17 [55, 56]. In humans, Th22 localizes to the skin and can express IL-22, TNF*α*, and IL-13 [57]. In human skin, the IL-22R1 subunit of the IL-22 receptor complex is constitutively expressed mainly by nonhematopoietic tissue resident cells; it was observed in keratinocytes and was upregulated by IFN*γ* [58]. In psoriatic patients elevated IL-22 plasma levels have been detected, which correlated with the disease severity. The role of IL-22 in psoriasis pathogenesis has been linked to keratinocyte activation and to the formation of epidermal acanthosis, a prominent morphologic feature of psoriasis [35, 59]. IL-22 indeed regulates keratinocyte function in multiple ways. In particular, it favors the formation of a biological barrier of the skin through the induction of antimicrobial proteins in keratinocytes, it inhibits terminal keratinocyte differentiation, it induces the production of matrix metalloproteinases 1 and 3 linked to tissue degradation, and it recruits neutrophils through the induction of chemokine production [59]. Together with other cytokines, such as TNF*α*, IL-17, and IL-20, IL-22 therefore takes part in the formation of the cytokine network that orchestrates the progression of the different pathogenic features of psoriasis [60].

An important study has reported that epidermal Th22 cells in resolved psoriatic plaques were still functional after several years of disease remission underlining a role for tissue resident Th22 in psoriasis pathogenesis and in disease memory in sites of recurrent psoriasis [11].

In mouse imiquimod models of psoriasis, IL-22-deficient mice have an evident decrease in the formation of psoriasis-like lesions, in particular pustule formation and acanthosis. Moreover, using an autoimmune psoriasis model, mice treated with IL-22 neutralizing antibody demonstrated either no development or extremely mild development of psoriasis, which suggests that IL-22 antagonism may lead to a therapeutic approach [58, 59, 61].

## 4. Th9 and Pathogenic T Helper Subsets

Th9 cells represent a skin-tropic subpopulation which is present both in healthy and in diseased human skin [62, 63]. Th9 cells have been detected within psoriatic skin lesions as well as in other inflammatory skin disorders such as atopic dermatitis. In psoriatic skin lesions, the number of IL-9-producing cells was found to be higher compared to healthy skin, and gene expression analysis indicates significantly augmented* IL9* expression as compared to normal skin from healthy subject [49, 62]. Th9 cells are found primarily among the CLA^+^ skin-homing T cell population. CLA^+^ Th9 cells upon activation can rapidly and transiently produce IL-9, which in turn exerts an autocrine effect by inducing further IL-9 production and a paracrine effect by inducing IFN*γ*, IL-17, and IL-13 production by CLA^+^ Th1, Th2, and Th17 cells. The ability of Th9 cells to enhance the production of inflammatory cytokines from other T cell subsets and their increased presence in psoriatic skin lesions suggest that Th9 cells may also participate in initiating and maintaining cutaneous inflammation [62].

Finally, a role for Th21 has been proposed in psoriasis pathogenesis [64]. IL-21 is indeed expressed at higher level in psoriatic skin lesions, where it is mainly produced by infiltrating CD4^+^ T cells and by NK cells. As IL-21R is expressed by both T cells and keratinocytes, it has been proposed that IL-21 could play a role in disease pathogenesis either by exerting a mitogenic effect on keratinocytes thus acting in synergy with factors such as IL-22 or by expanding other pathogenic T helper cell subsets. Noticeably, IL-21 was also found to be expressed by T cells coexpressing IFN*γ* or IL-17. This evidence brings us to the concept of specific pathogenic T helper subsets in the pathogenesis of psoriasis. Pathogenic T helper cells are now defined as subsets of T cells that develop from classical Th1/Th2 and Th17 subsets under specific conditions. They secrete a distinguishing cytokine profile and possibly exert a role in the induction of the diseases [65]. In psoriatic skin lesions, a subset of cells coexpressing IL-17 and IFN*γ* has been described, which can possibly fall into the recently defined categories of non-classic Th1 cells or Th1^*∗*^ cells [27, 66–68]. These cells have more aggressive inflammatory phenotype and they may be regarded as an example of disease-inducing T helper subset.

## 5. Emerging Role of Recirculating T Helper Cells in Different Clinical Manifestations of Psoriasis

Besides skin and joint inflammation, there is now evidence that psoriasis is associated with important systemic manifestations and comorbidities such as cardiovascular disorders, metabolic syndrome, and Crohn's disease [5, 6].

As an explanation to the systemic manifestation of the psoriatic diseases, the concept of “psoriatic march” has been introduced, which views psoriasis as a state of systemic inflammation that leads to the involvement of other organs besides skin through the systemic circulation [6, 69].

In this process, soluble mediators of systemic inflammation have been identified and include serum TNF*α*, vascular endothelial growth factor (VEGF), IL-12, monocyte chemotactic protein-1 (MCP-1), S100A8/A9, and importantly systemic levels of circulating IL-17A of particular relevance for cardiovascular comorbidities [5].

Psoriasis patients with moderate-to-severe disease have significantly elevated serum IL-17A levels [30, 70], which appear to be associated with higher risk of developing cardiovascular complications including stroke and myocardial infarction. In human atherosclerotic lesions, IL-17A/IL-17F levels are increased at each stage of plaque development and in advanced coronary plaques CD4^+^ T cells expressing both IFN*γ* and IL-17 were also detected [71, 72].

Despite this evidence, the cell trafficking events representing the pathogenic link between the skin manifestations and systemic inflammation and development of comorbidities are still largely unknown.

In psoriasis patients, we have previously described the presence of dermal lymphoid aggregates mainly composed of CD4^+^ T cells and myeloid dendritic cells. CD4^+^ T cells in these aggregates expressed CCR7 and the axis CCR7/CCL19 in psoriatic skin lesions was shown to play a critical role in the disease pathogenesis and in disease remission induced by TNF-blockers [4].

In a Kaede transgenic mouse model, Luster and coworkers have demonstrated that CD4 memory T cells can exit from the skin in a CCR7-dependent manner. A subpopulation of recirculating memory CD4^+^ T cells in peripheral blood with a CCR7^+^CD69^−^CD103^+/−^CCR4^+/−^ phenotype has been identified, which can enter the circulation and maintains the ability to migrate into normal skin [73].

It is therefore possible to hypothesize that human diseases of barrier tissues, such as psoriasis, may involve a similar dynamic balance between tissue resident memory T cells and the recirculating pool [74].

Our recent data indicate that specific subsets of memory T cells are involved in the clinical manifestations of cutaneous psoriasis and in systemic inflammation. In particular, we identified that CCR5^+^ CD4^+^ T cells are inversely associated with the extent of the cutaneous manifestations, suggesting a contribution in the formation of the psoriatic plaque [49]. By contrast, we observed increased circulating percentage of CCR4^+^ T cells in psoriasis patients. CCR4^+^ CD4^+^ T cells with T_EM_ and T_eff_ phenotype in the circulation correlated positively with the severity of the cutaneous disease; however, we did not find any significant correlation with systemic inflammation.

CCR4 has been defined as a skin-homing receptor that is upregulated by memory T cells which have been primed in skin draining lymph nodes. T cells expressing CCR4 can therefore relocalize to the skin when inflammatory signals induce the expression of its ligand CCL17 [75–77]. These observations suggest that CCR4^+^CD4^+^ T cells may represent a subset of cells which recirculates from the skin with the ability to relocalize to the skin compartment upon antigen reexposure or expression of inflammatory signals [76, 77].

By contrast, we found that CCR4^+^ CD8^+^ CD103^+^  T_eff_ cells in the circulation highly correlated with systemic inflammation and with PASI score, thus representing a possible link between the severity of the cutaneous manifestations of the disease and systemic inflammation.

In the CD4 compartment, we found that CCR6^+^ CD4^+^  T_EM_ cells correlated with systemic inflammation measured as serum level of C-reactive protein (unpublished data), in line with the increase of IL-17A in serum of patients with severe psoriasis and with its association with cardiovascular diseases.

This initial evidence supports that T helper cell recirculation from the skin can play a role in either the amplification of the cutaneous manifestations or in the development of comorbidities associated with psoriasis. Further investigations could possibly provide additional information elucidating the connection between T cells responses arising in the skin and the systemic manifestations of psoriasis.

## 6. Conclusions

Different subsets of helper cells have been reported to contribute to the pathogenesis of psoriasis. However, it is important to define for each subset the role in the psoriatic cascade and in the clinical manifestations of the disease. In particular, IL-23/Th17 axis was shown to be central in amplification of inflammation and cutaneous manifestations of psoriasis as supported by the high therapeutic efficacy of anti-IL-17 antibodies used in clinical trials. By contrast, Th1 cells likely participate in earlier stage of psoriasis pathogenesis by inducing CCL20 and IL-23 production by myeloid dendritic cells, thus playing a role upstream of the proinflammatory cascade controlled by IL-23/Th17 axis.

Tissue resident Th22 together with CD8 Tc17 cells have been proposed as repository of disease memory in sites of recurrent psoriasis and because of the action of IL-22 on keratinocytes, they are linked to the most prominent histological features of psoriasis. Recently, IL-9-producing Th9 cells have been found to play significant roles in the pathogenesis. Finally, there is an emerging a role for CD4 T cells recirculating between skin and blood in the clinical manifestations of psoriasis and the associated comorbidities. In particular, possible roles for CCR4^+^ CD4 and CD8 T cells in amplification of skin and in systemic inflammation have been indicated, respectively.

Further investigations are needed to elucidate the connection between T cells responses arising in the skin and the comorbidities associated with psoriasis.
